# Inequities towards leprosy-affected people: A challenge during COVID-19 pandemic

**DOI:** 10.1371/journal.pntd.0008537

**Published:** 2020-07-24

**Authors:** Sharika Mahato, Srijana Bhattarai, Rakesh Singh

**Affiliations:** 1 Monitoring and Evaluation Department, The Leprosy Mission Nepal, Kathmandu, Nepal; 2 School of Public Health, Patan Academy of Health Sciences, Lalitpur, Nepal; 3 Department of Community Health Sciences, School of Medicine, Patan Academy of Health Sciences, Lalitpur, Nepal; Hospital Infantil de Mexico Federico Gomez, MEXICO

On January 30, 2020, following the recommendations of the Emergency Committee, the World Health Organization (WHO) declared the outbreak of the 2019 coronavirus disease (COVID-19) as a Public Health Emergency of International Concern [1]. As the COVID-19 pandemic rages across the countries, burden of COVID-19 has reached to 215 nations in the world by June 19, 2020, with 8,750,990 cases, 461,820 deaths, and 4,620,378 recoveries according to Worldometer COVID-19 data. Of that, 20.75% of the cases and 10.04% of the deaths have been reported from Asia. While India alone accounted for 395,812 cases and 12,970 deaths, Indonesia reported 43,803 cases and 12,970 deaths, and Nepal ranked 25th among Asian countries with 8,274 cases and 22 deaths due to COVID-19. The increasing burden of COVID-19 accompanied by travel restrictions, lockdown measures, and restrictions on social gatherings and activities have disrupted several lives. Not only a health crisis has been brought by the COVID-19 pandemic: It is affecting societies and economies at their core and will most likely increase poverty and inequalities at a global scale, making achievement of Sustainable Development Goals (SDGs) even more urgent [2]. Moreover, the nationwide lockdown accompanied by travel restrictions have been threatening all citizens, with greater impact on vulnerable social groups (including those affected with leprosy) for accessing healthcare services in particular.

People of all ages can be infected by COVID-19. However, the elderly and people with preexisting medical conditions appear to be more vulnerable to becoming severely ill with the virus, according to WHO [3]. Leprosy is a mycobacterium infectious disease affecting peripheral nerves. Leprosy-affected people with leprosy-related reactions could be one of the conditions for being at high risk of COVID-19 infection [4].

Worldwide, there are 23 global priority countries for leprosy as per its burden. As per data in 2019, 79.6% of the new leprosy cases are concentrated in India, Brazil, and Indonesia. Countries of the South East Asian Region (SEAR) constitute 71% of the global new cases, with India and Indonesia attributing 92% of the case burden in the region [5]. Despite the declaration of leprosy elimination in 2010, the prevalence of leprosy increased from 0.77 in 2010 to 0.83 in 2014 in Nepal [6]. Nepal is among the global priority countries of leprosy in SEAR, with an increase in new cases from 3,054 in 2016 to 3,249in 2018, an increase in new grade 2 disability (G2D) cases from 87 in 2017 to 133 in 2018, and an increase in pediatric cases in 2018 [5].

Leprosy can be cured through treatment via drugs, and the treatment in the early stages can prevent disability among the leprosy affected people [7]. However, if remained untreated, leprosy can cause progressive and permanent damage to the skin, nerves, limbs, and eyes [7]. This makes early diagnosis of leprosy an efficient way of treatment,as the damages are irreversible. Even after treatment, many patients are left with deformities, sores, cracked skin, and amputated limbs [8].

For leprosy affected people, COVID-19 has a massive impact because (in places with fragile health systems like Nepal and India) all the nonurgent hospital consultations and admissions are being discouraged in health facilities because of concerns of responding to increasing COVID-19 cases [4]. This has shifted the hospital priority toward COVID-19 responses, putting other health conditions at least priority at the moment. This situation has created a gap in health service needs for leprosy patients. Although a number of satellite clinics have been established to provide special leprosy services at community levels [9], this as well has been affected by the COVID-19 pandemic. Moreover, not only the access to the hospital has been an issue for leprosy affected persons, but maintaining personal hygiene on their own is another major challenge for them. Frequent hand-washing, being one of the essential ways of preventing COVID-19 transmission, could be one of the biggest challenge for leprosy patients with deformities, cracked and dry skin making them unable to maintain the personal hygiene [8]. Leprosy as a disease of poverty would affect the patients' availability for soap and sanitizers for hand washing [10], increasing their risk to contract COVID-19.

Leprosy affected people can suffer from two types of autoimmune reactions. Type 1 leprosy reaction or reversal reaction is presented as a delayed hypersensitivity reaction seen in borderline leprosy, manifesting edema, erythema, and neuritis. Neural impairment is important in the clinical context of reversal reactions, and it is the leading cause of death and deformities. The drug of choice is corticosteroid therapy,but they suppress inflammatory immune response through interference with the activation of immune cellular response due to which immunosuppressive doses of corticosteroids should be maintained for a long period [11]. Furthermore, the study has shown important actions of low dose methotrexate in the leprosy patient intolerant to corticosteroid therapy [12]. Type 2 leprosy reactions, erythema nodusumleprosum (ENL) is seen in patients with borderline lepromatous and lepromatous leprosy, which manifest sudden appearance of skin papules, erythematous plaques, erythematous, and painful hard lumps distributed throughout the body. Type 2 reactions can be treated with the 6-months therapy of thalidomide. However, if treatment with thalidomide is continued over a longer period, there is a danger of neuropathy that can be severe and irreversible [11]. The drugs that are already been used in the leprosy reactions with proven efficacy are being tested in the COVID-19 patients, with increasing evidence of using thalidomide or steroids, including dexamethasone [12]. The leprosy patients, if infected with COVID, might be protected from the effect of COVID-19. However, the long-term use of corticosteroid and immunosuppressant therapy itself has its effect and increases its susceptibility to COVID-19.

Leprosy has been associated with social stigma and is still present in communities of Nepal [6], and the affected people have been facing stigma, leading to social exclusion and poor mental well-being [7]. The deep-rooted stigma of leprosy in the communities has been an underlying cause of social inequities faced by the people affected with leprosy, which in turn has been threatening their health and livelihood. Now, existing social distancing recommendations as public health measures to the COVID-19 pandemic may worsen already marginalized populations, including leprosy-affected people. The measures towards the COVID-19 pandemic, including social distancing and travel restrictions, are threatening the psychological well-being because of the attached stigma, discrimination, loneliness, and isolation [13]. Leprosy already being a stigmatized condition, these measures have been affecting people with leprosy even more, with their limited social network and disrupted lives. To tackle the stigmatized condition and its risk towards psychological well-being, self-help support groups, peer counseling, and community-based individual care are among the most appropriate interventions [14]. However, actions such as social distancing and travel restrictions hinder these social interventions during the pandemic. Hence, the barriers for accessing services affecting persons with leprosy are exacerbated during this pandemic. Furthermore, this may lead to increased loneliness among them, which may further affect their anxiety and depression level, combined with additional fear of COVID-19 infection [15]. The recently developed federal system of Nepal is ill prepared for outbreaks and lack coordination [16] and has been facing difficulty in mitigating the impacts of the pandemic. In this condition, even after the normalization of the pandemic situation, the resume in health services will take a longer time due to limited resources and the fragile healthcare system.

The COVID-19 pandemic has revealed an urgent need to address gaps in the country’s public health infrastructure. In fact, this pandemic has unveiled underlying structural vulnerabilities and existing health and social disparities in many settings, including the big economies such as the US and Europe. Many in leprosy control have focused entirely in developing neoliberal interventions, such as vaccines or drugs, that may act as silver bullets for a disease such as leprosy (and also COVID-19) that is closely connected with human behavior and insufficient sanitation. Moreover, the underlying social inequities that led to the occurrence of leprosy at this point in the history of mankind has now been exacerbated in the form of similar underlying forces, leading to the disproportionate impact of COVID-19 among the marginalized members of society, including those affected with leprosy. As the underlying roots of leprosy are completely social, zero discrimination has been identified as a possible indicator and target for the post-2020 global leprosy strategy [5]. However, the major focus in the global fight towards leprosy has been put towards other dimensions including raising funds for case finding and treatment intervention. Nevertheless, while early case finding and prompt treatment are keys towards controlling leprosy, interventions addressing social determinants of leprosy are equally important in the global fight towards the social burden of the disease.

In conclusion, to truly be able to eliminate or eradicate leprosy (highly questionable), there is an absolute need for addressing the underlying social structures that translate into food insecurity, housing insecurity, lack of education, living in poor sanitary conditions, malnutrition or poorly nutritional diets, and living lives with more dignity. The solution is not a vaccine but is social improvements in the lives of those affected with leprosy or atrisk of becoming infected. Furthermore, creating an enabling environment for continuation of leprosy-related healthcare services should be a priority in countries like Nepal during public health crisis such as the COVID-19 pandemic.
